# Artificial intelligence in coronary artery calcification scoring: Current progress and future directions

**DOI:** 10.21542/gcsp.2025.42

**Published:** 2025-08-30

**Authors:** Mobin Ghaderi, Reza Golchin Vafa, Najmeh Vosoughiyan, Sultan Mujib Dabiry, Omneya Abdelkarem

**Affiliations:** 1 Student Research Committee, Kurdistan University of Medical Sciences, Sanandaj, Iran; 2 Cardiology Department, Shiraz University of Medical Science, Shiraz, Iran; 3 Yüksek İhtisas University, School of Medicine, Ankara, Türkiye; 4 Chemical Pathology Department, Medical Research Institute, Alexandria University, Egypt

## Abstract

Objective:  The primary purpose of this paper is to evaluate the role of artificial intelligence (AI) in enhancing coronary artery calcification (CAC) scoring for improved cardiovascular risk assessment.

Methods: A narrative review was performed using data from PubMed, Scopus, and Semantic Scholar, focusing on publications from 2020 to 2025. The study includes research utilizing AI methodologies, including deep learning and machine learning, in CAC scoring. Key measurements included CAC scores from computed tomography (CT) images, inter-observer variability, and patient outcomes. Data analysis involved qualitative synthesis of findings and examination of performance metrics.

Results: AI algorithms significantly improved CAC score accuracy, with sensitivity and specificity rates of 90%. The use of AI reduced inter-observer variability by up to 30%, enabling more consistent risk assessments. Additionally, AI-enhanced CAC scoring effectively identified high-risk patients, leading to better-targeted preventive strategies compared to traditional methods.

Conclusion: The incorporation of AI into CAC scoring holds promise for transforming cardiovascular risk assessment by enhancing accuracy and reliability. Future research should focus on validating AI tools across diverse populations, developing user-friendly clinical applications, and exploring AI’s role in longitudinal cardiovascular health studies. Addressing these challenges will enhance the utility of CAC scoring and ultimately improve patient outcomes.

## 1. Introduction

Coronary artery disease (CAD) is the most common type of cardiovascular disease and a significant cause of mortality worldwide^1–3^. Statistics indicate that approximately 17.9 million individuals lose their lives to this disease annually^4^. Risk assessment is essential for the early identification and effective management of coronary artery disease. This evaluation requires cost-effective, non-invasive, and available methods^1^. One of the newer methods to improve traditional risk assessment approaches involves diagnosing coronary artery calcification (CAC) through CT scan images^5^. Coronary artery calcification occurs when atherosclerotic plaques are deposited. The degree of calcification in coronary arteries is directly related to the extent of atherosclerosis^6^. Computed tomography (CT) scans serve as a dependable metric for assessing the extent and progression of atherosclerosis in arteries^7^. The coronary artery calcium scoring (CACS) model is a method for measuring calcium deposits on the coronary artery wall via CT imaging. Research has demonstrated that CACS is a powerful tool for predicting cardiovascular events and is closely associated with cardiovascular risks across different races regardless of age, gender, or other risk factors^8,9^. Coronary artery calcium scoring is one of the most reliable risk indicators of atherosclerotic cardiovascular disease (ASCVD) in asymptomatic individuals^10^. In addition, with the progression of coronary artery calcium burden, the likelihood of cardiovascular adverse events also increases^11^. This measurement can be performed using electron beam CT or multi-detector CT^7^. This method provides a clearer view of coronary plaque burden and allows for risk assessment of individuals^12^. CACS can enhance the accuracy of risk prediction and deliver better results than standard algorithms based on traditional risk factors^5^.

Despite the clinical importance of CAC, its application and use encounter several limitations^13^. CAC scoring with standard methods using specialized cardiac CT scans has limitations such as the necessity for ECG gating, contrast agents, and significant expenses^14^. The CAC scoring model using gated coronary CT scan imaging requires considerable resources and equipment such as capital-intensive CT machines, which not all centers may be able to provide^15^. It is also challenging to assess very low and nearly undetectable calcium levels with currently available diagnostic methods^13^. Moreover, examining slice-by-slice CT scan images is a time-consuming task, which leads to delays in reporting CAC scores due to personnel shortages and may also result in inaccurate reports. Additionally, many patients undergo routine chest CTs for various reasons such as screening for lung cancer, infection, etc., and their CAC scores are often not recorded, which may lead to missed opportunities for patient treatment or prevention. Applying artificial intelligence and enhancing measurement techniques may reduce these limitations^15,16^ and help stratify patients more effectively^13^.

However, even with these technological advances, several key limitations remain unresolved. First, current AI approaches face challenges related to variability in data quality, lack of large-scale diverse training datasets, and inconsistent performance across different imaging protocols and patient populations, limiting their generalizability and reliability in clinical practice^17,18^. Furthermore, conflicting findings have been reported in the literature regarding the accuracy and clinical utility of AI-based CAC scoring, reflecting methodological heterogeneity and limited real-world validation. Importantly, available evidence is preliminary, and AI-based tools should be viewed as complementary to, rather than substitutes for, expert clinical judgment at this stage^19^. Future research should focus on standardized validation of AI models across diverse populations and integration into clinical workflows, with transparent reporting of limitations to avoid overestimating their current capabilities^20^.

Recent advances in artificial intelligence (AI), especially in deep learning (DL) employing convolutional neural networks (CNNs), have shown considerable potential in medical imaging^21^. Artificial intelligence is utilized in cardiovascular health to identify new diseases, estimate patient risks, reduce treatment costs, and more^22^. Using AI in cardiac radiology can help physicians better manage patients who may have coronary artery disease. It assists in diagnosis, prognosis, and predicting disease progression over time^23^. Machine learning algorithms have shown equivalent or superior performance accuracy compared to humans in heart disease diagnosis, prediction, and other medical procedures^24^.

Machine learning algorithms can surpass traditional scoring models and methodologies and achieve better outcomes based on clinical variables and radiographic images^25^. Artificial intelligence algorithms can identify different levels of risk in patients with cardiac conditions by evaluating CAC data. Advances in AI have generated a new approach for identifying coronary artery calcium that could improve cardiovascular health assessment^26^. Additionally, the measurement of CAC with artificial intelligence can transform cardiovascular risk classification and provide the possibility of earlier diagnosis and more comprehensive and accessible screening^27^. AI not only simplifies and accelerates the time-consuming process of measuring calcium but also allows collection of CAC scores from routine chest CT scans performed for other indications. This includes low-dose CT, cardiac CT angiography, and PET/CT^28^. Given the advantages of adopting artificial intelligence in CAC score classification, expenses, human resources, and time can be saved through AI implementation^29,30^. In light of existing evidence, we undertook this research to investigate the effect of employing artificial intelligence in the evaluation of coronary artery calcification, enhancing diagnostic accuracy and access to medical services while decreasing costs and diagnostic time. Considering the limitations of traditional imaging and scoring methods, this study examines how artificial intelligence can improve existing diagnostic procedures and help clinicians manage patients more effectively.

## 2. Methods

We conducted a narrative literature review to explore the role of artificial intelligence in coronary artery calcium (CAC) scoring. The literature search was performed in PubMed (MEDLINE), ScienceDirect, and Semantic Scholar databases. We used the following search terms and Boolean operators: (“Artificial Intelligence” OR “AI” OR “Machine Learning” OR “Deep Learning”) AND (“Coronary Artery Calcification” OR “Coronary Calcium Scoring” OR “CACS”). Relevant studies published between January 1990 and March 2025 were included. Non-English publications, editorials, letters, and conference abstracts without full text were excluded from the study.

## 3. AI in image acquisition and preprocessing

Over the past decade, artificial intelligence (AI) employing machine learning (ML) and deep learning (DL) has demonstrated considerable advancements in medicine, encompassing cancer diagnosis, retinal disease assessment, and medical image analysis^31–34^. In radiology, the primary branches of AI encompass machine learning for analyzing complex patterns in imaging data, deep learning for enhancing image interpretation and streamlining workflow, and natural language processing (NLP) for assisting report generation and supporting clinical decision-making^35^. Radiology data, including textual reports and archived images, provides an appropriate foundation for machine learning and is thus regarded as an optimal platform for the development of AI tools. This has resulted in the advancement of research into AI applications in radiology^36^.

Well-trained AI models can analyze medical images within seconds and deliver accurate analytical results. Studies have shown that these models have comparable performance to medical experts in some cases and can even provide superior results in disease diagnosis. Despite high accuracy, the notable benefits of AI in cost reduction and enhanced speed substantially influence the improvement of clinical diagnosis, treatment, and prognosis while reducing the workload of both specialists and patients^37^. In this regard, advanced deep learning algorithms play an important role in medical image analysis and assist in early disease diagnosis and the development of treatment approaches^38,39^. This technology assists radiologists by streamlining processes such as image interpretation, anomaly identification, and clinical decision support. Consequently, the time required for standard procedures is reduced, allowing specialists to focus on complex cases^35^.

Convolutional neural networks (CNNs) are one of the powerful subsets of AI models that have made substantial advances in medical image processing. These models play a vital role, particularly in the field of computer vision, which allows devices to “see” and analyze visual input. The adoption of this technology enhances the accuracy, speed, and accessibility of medical image analysis^40^. One prominent example of AI’s potential in radiography is the detection of coronary artery calcification. AI-based solutions have shown promise in automating the diagnostic process, aiding in early diagnosis, and improving patient outcomes. AI algorithms can achieve accuracy equivalent to or even superior to that of expert radiologists^41^.

## 4. AI for automated CACS quantification

Artificial intelligence involves creating hardware and software that can perform tasks associated with human intelligence^42^. AI is rapidly transforming medicine by helping physicians make more accurate diagnoses, personalizing treatments, and automating repetitive tasks^43,44^.

To provide a basic understanding and brief explanation of how AI is used in medical imaging, here is an overview of some key concepts. However, a detailed explanation of the various forms of AI can be found in the review by Gennari et al. ^45^:

∘ Machine learning (ML) involves training algorithms to learn patterns from data, improving their performance over time^46^.
∘ Deep learning (DL) is a subset of machine learning that uses multiple layers of artificial neural networks (ANNs) to extract complex features from both structured and unstructured data^47^.
∘ Convolutional neural networks (CNNs) are specialized ANNs designed for image processing, particularly skilled at identifying and segmenting objects in medical images^47^.

AI approaches medical data analysis very differently from traditional rule-based systems, which use human-programmed predetermined rules. Traditional rule-based systems rely on predefined rules programmed by humans based on existing medical knowledge and struggle with the complexity of medical data^45^. AI, specifically ML, does not rely on fixed or predetermined rules; rather, ML algorithms directly learn correlations and patterns from large datasets, identify complex patterns, and make predictions based on the data on which they are trained^46^. Table 1 summarizes how AI’s capacity for learning and adaptation makes it ideal for evaluating medical data.

**Table 1:** Comparative analysis of traditional rule-based systems and machine learning in terms of analysis basis, adaptability, complexity handling, and human intervention.

**Feature**	**Traditional Rule-Based Systems**	**Machine Learning**
**Basis of Analysis**	Pre-defined rules by humans. Involves human-designed algorithms where specific instructions and rules are programmed to identify and quantify calcifications	Patterns learned from data. AI, uses computers to replicate human cognitive functions like interpreting structured data such as medical images. Deep learning models are particularly effective at processing visual information and learning features from vast datasets.
**Adaptability**	Limited, requires manual updates and interventions. For semi-automated methods, users (radiological technologists, cardiologists, or radiologists) must manually decide to adopt or reject each candidate calcification.	Adaptive, improves with more data. Algorithms can be retrained with additional data to improve performance. New areas of exploration are emerging that can integrate different approaches as data accumulates.
**Complexity Handling**	Struggles with complex patterns. Coronary calcification can be heterogeneous, meaning scoring from a specific vessel may underestimate true risk, and traditional methods struggle with this variability. Semi-automated methods can be complicated and include false candidates like pleural or pericardial calcification.	Can handle complex relationships. Deep learning systems can accurately predict cardiovascular events by quantifying the presence and extent of coronary calcium. They can identify associations between complex input variables (medical images) and outcomes.
**Human Intervention**	High, requires constant programming. Time-consuming or complicated measurements are often omitted in busy clinical settings. Training of personnel is mandatory to reduce decision errors in semi-automated quantification. Prone to interrater interpretation variability.	Lower after initial training, leading to fully automated processes. AI can free clinicians from tedious and repetitive tasks. The system can calculate the calcium score in under 2 s without human input, making it an ”end-to-end” solution. However, human backup and review of AI results are still important. AI is also being used to reduce radiation doses and improve image reconstruction.
**Accuracy & Agreement**	Susceptible to measurement variability depending on the software and the experience of the operator. Even minor scoring inaccuracies can result in substantially different risk stratifications.	Has shown overall efficacy and a high level of agreement with categorization by trained clinicians. Automated systems have shown excellent correlation and agreement with semi-automatic software for various CAC scores. The pooled kappa statistic of 83% indicates strong agreement between automated and manual CAC scoring.
**Computational Speed**	Time-consuming for manual/semi-automated scoring. For segment-level scoring, median annotation time was 1.85 min per scan.	Significantly faster. Mean computational time ranged from 2 s to 10 min. The deep learning system can assess CAC scores in under two seconds per scan. This automation may reduce costs and streamline workflow.
**Transparency**	Generally clear how conclusions are reached (human-driven process).	Can involve a ”black-box problem” where it is unclear how the AI reaches a conclusion due to the complexity of neural networks. Decisions may need to be made between easily interpretable but less accurate models versus more accurate but less transparent ”black-box” models.

### The role of artificial intelligence in evaluating coronary artery calcium score (CACS)

Clinicians are increasingly turning to artificial intelligence (AI) to tackle one of cardiology’s most time-consuming tasks: calculating coronary artery calcium scores (CACS). For example, AI tools can significantly accelerate the processing of CT scans for CACS and potentially identify subtle calcifications that might be missed by human readers, not only expediting diagnoses but also detecting subtle calcifications that even experienced radiologists might miss. How reliable are these AI systems? This section explores the techniques behind them, particularly deep learning (DL) and convolutional neural networks (CNNs), while also considering issues such as overfitting and bias in datasets. CNNs have gained traction in medical imaging because they function similarly to human vision, analyzing images in layers. By identifying specific features, such as calcified plaques in coronary arteries, they have transformed CACS quantification. However, their “black-box” nature raises concerns; a 2023 study found that CNNs occasionally misclassify artifacts as calcifications, especially in low-resolution scans. Studies highlight their role in minimizing human error during analysis, though challenges such as dataset bias and generalizability remain unresolved^45,46^.

**Figure 1.**
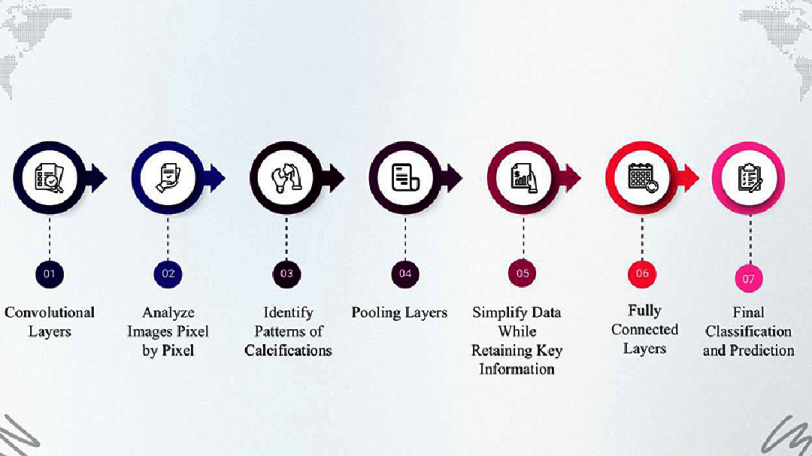
This flowchart visually represents the stages of processing CACS using CNN.

CNNs rely on three core layers to process medical images^48^:

1. Convolutional layers: Think of these layers as the AI’s “magnifying glass.” They analyze CT images one pixel at a time, searching for patterns such as the subtle, dotted calcifications in coronary arteries that even skilled radiologists may overlook. They are designed to identify calcifications based on their high density, typically above 130 Hounsfield units (HU). But there is a limitation: they can sometimes mistake image noise (such as motion artifacts) for actual calcifications.
2. Pooling layers: These layers simplify the data without losing essential information. For example, they will reduce a feature map highlighting calcification density but keep the core information intact, similar to compressing a high-resolution photograph into a smaller file without erasing key details. However, over-aggressive pooling can blur subtle calcifications, leading to underestimated CACS values.
3. Fully connected layers: This is where the integration occurs. These layers take the filtered features, such as the size and density of a calcified plaque, and make a final classification or prediction. However, perfection should not be assumed; if the earlier layers misfire (e.g., miss a calcification), these layers amplify the error, skewing risk predictions (Figure 1).

Additionally, researchers have developed several architectures to address specific challenges in CACS assessment, which specialize in segmentation, classification, and risk prediction:

• U-Net: First introduced by Olaf Ronneberger in 2015^49^, its “skip connections” were an innovation for segmenting small calcifications in coronary arteries. However, there is a limitation regarding its accuracy, which decreases with noisy or poorly annotated training data^50^. In a study, the U-Net model achieved a micro-average F1-score of 0.581 at the lesion level, which is lower compared to other models^15^.
• U-Net++: To address U-Net’s limitations, Zhou et al. added nested skip pathways in 2018, improving boundary detection in CAC measurements^51^. The tradeoff? Hospitals often lack the computational power to run U-Net++ efficiently, a limitation highlighted in a 2022 meta-analysis^52^.
• 3D U-Net: Unlike its 2D predecessor, this version analyzes CT scans in three dimensions, leveraging adjacent slices to better map calcification across coronary arteries. While promising, its adoption is limited by the lack of annotated 3D datasets. Recently, a 3D U-Net model was trained on a database of 783 CT examinations and achieved a C-index of 0.951 on the testing set^53^.
• ResNet/VGGNet: These networks are used in classification tasks in CACS, as they help categorize the severity of calcification and predict cardiovascular risk. VGGNet uses small filters to increase CNN depth^50^, while ResNet’s “residual blocks” solve the degradation problem and prevent accuracy loss in deeper network architectures^54^.

### 4.1. Evaluation and verification of AI in CACS

Recent studies show that AI provides high accuracy and reliability in coronary artery calcium scoring (CACS)^53^. Intraclass correlation coefficient (ICC)^55,56^ and Cohen’s kappa statistics show strong agreement in assessment between AI-generated and clinician-derived coronary artery calcium scoring. However, some challenges persist in technical and practical domains. High ICC values, such as 0.996 for the Agatston score, 0.988 for total CACS, and 0.993 for test-retest reliability, indicate strong agreement between automated and manual scoring^56,57^. Cohen’s kappa is particularly appropriate for CACS as it measures interrater agreement for categorical items, accounting for chance. It assesses how closely automated deep learning models replicate human expert scoring, thereby quantifying the reduction in interrater variability and standardization^58^. Studies report a weighted Cohen’s kappa of 0.808 for segment-level agreement and 0.909 for risk category assignment, with a pooled kappa statistic of 83% across 25 studies indicating strong agreement between automated and manual CACS. Other significant metrics include the F1-score, which is the harmonic mean of recall and precision, reported as 0.90 for calcified volume and 0.87 for the number of calcified lesions, along with sensitivity (e.g., 0.94 for proximal RCA) and specificity (e.g., 0.978 for segment-level agreement), all of which underscore the efficacy of AI in detecting and localizing calcifications^59^.

### 4.2. Challenges in AI-based CAC detection and segmentation

Several challenges exist in the development and implementation of AI-based CAC detection and segmentation, stemming from technical and practical considerations. One significant issue is the heterogeneity of CAC, which can be localized in specific vessels or plaques, or distributed across various coronary arteries. This variability complicates the task for AI to accurately identify and quantify CAC. Additionally, these algorithms may struggle to differentiate CAC from other high-density structures such as pleural or pericardial calcification and the calcifications found in the aorta and cardiac valves^45^.

Image quality is another critical factor; scanner types, imaging protocols, noise, and patient-specific factors (body size and motion artifacts), particularly in non-ECG-gated images, hinder precise CAC detection^45^. The success of AI algorithms also depends on the quality and diversity of training datasets, which ideally should encompass different scanning platforms and diverse populations. Inadequate diversity can result in skewed outcomes. However, the creation of these datasets is both time-consuming and expensive, which presents a significant challenge in developing reliable AI algorithms^60^.

Segmentation of CAC presents additional difficulties due to the low contrast between coronary arteries and the myocardium in non-contrast images. Cardiac motion artifacts in non-gated CT images can also impede segmentation methods. Additionally, the “black-box” problem, which makes it difficult to understand how AI arrives at a conclusion, presents a significant challenge. Many AI models, particularly DL models such as CNNs, act as a “black box.” This lack of transparency in understanding how an AI model arrives at its conclusion undermines clinicians’ trust in AI-based CACS systems^29^.

On the practical side, integrating AI into existing clinical workflows poses a challenge that requires careful planning. It is essential to determine whether AI will assist in clinical decision-making at the point of care, serve as part of a risk dashboard, or simply flag patients for further evaluation^58^. Moreover, the absence of standardized methods for training, validation, and reporting AI performance limits the ability to compare different studies, which is compounded by the need for human oversight^29^.

Collaboration between computer scientists and clinicians is vital for the effective development and implementation of AI-based CAC scoring; however, this collaboration has been insufficient. Clinicians must feel confident that AI will enhance their work rather than replace them. Reproducibility is also a concern, as different AI algorithms may yield varied results when applied to the same dataset due to specific characteristics related to the scanner, field of view, reconstruction filter, and slice thickness^45^. Finally, many AI models still lack external validation necessary to confirm their effectiveness in new environments.

### 4.3. ECG-gated vs. non-ECG-gated CT imaging in AI-driven coronary artery calcium scoring

Electrocardiogram (ECG)-gated and non-ECG-gated CT imaging are two different methods for acquiring CT scans, particularly when evaluating the heart, and they have different implications for CACS. While both imaging methods can be used to assess CAC, they differ in several aspects, including image acquisition, motion artifacts, image quality, radiation exposure, and cost^45^. These differences are summarized in Table 2.

**Table 2:** Comparison of ECG-gated and non-ECG-gated CT scans for coronary artery calcium (CAC) measurement.

	**ECG-gated CT scans**	**Non-ECG-gated CT scans**
**Image Acquisition**	Synchronized with the cardiac cycle, usually during diastole (e.g., 65% or 75% of RR-interval). This prospective triggering aims to capture the heart at a specific, less-motion-prone phase.	No synchronization with the cardiac cycle
**Motion Artifacts**	Reduces artifacts, providing clearer images of the coronary arteries. The synchronization minimizes blurring caused by heart movement, crucial for precise CAC quantification.	More susceptible to motion artifacts, making CAC detection challenging. Cardiac motion can lead to an underestimation of the Agatston score. Algorithms designed for non-gated images have been developed to overcome this.
**Image Quality**	High precision. The Agatston score was originally developed based on ECG-gated images.	Low precision for direct Agatston score calculation compared to gated scans. However, AI methods can adapt well to novel CT types, including non-gated scans. Deep learning can be used to achieve comparable accuracy to manual scoring on non-ECG-gated images.
**Radiation and Cost**	More radiation, higher cost due to dedicated cardiac scanning protocols.	Less radiation, cost-effective as it utilizes scans already performed for other reasons (e.g., lung cancer screening, macrovascular diseases, routine medical check-ups).
**Clinical Utility**	Standard for CAC quantification and a cornerstone of contemporary cardiovascular imaging. Widely used as a predictor of cardiovascular risk.	High potential for broader ASCVD risk assessment in a wider population. A meta-analysis showed high correlation between scores from ECG-gated and non-gated images. AI-based methods can enable reliable and accurate CAC evaluation and risk stratification from non-ECG-gated chest CT.
**AI Performance**	AI-calculated Agatston scores show mean differences of −2.86 to 3.24 (older algorithms) and 0.0 to 1.3 (recent ones) compared to manual scores.	ICC values of 0.99 (chest CT) and 0.90 (low-dose chest CT for LC screening) have been reported for AI-based CACS quantification. AI and expert CACS evaluation showed good correlation on Bland-Altmann plots.

Despite the higher radiation levels associated with ECG-gated CT imaging, it is still considered the gold standard for CACS because it provides clearer images of the coronary arteries and facilitates precise quantification of calcium^61,62^. Although CACS was originally developed with ECG-gated images using semi-automatic quantification of calcified plaques based on 3-mm images by Agatston et al.^63^, a meta-analysis of 661 adults found a strong correlation between scores from ECG-gated and non-ECG-gated, non-contrast images^62^. Non-ECG-gated CT imaging is widely used for routine screening, such as for macrovascular diseases, pneumonia, and other lung diseases. This approach allows for the evaluation of CACS without exposing patients to additional radiation^61,64^.

### 4.4. Evidence for the accuracy and reliability of AI-based CACS quantification

AI-driven methods for coronary artery calcium scoring (CACS) are revolutionizing cardiovascular risk assessment, showcasing impressive accuracy and reliability compared to traditional expert scoring. Systematic reviews reveal a high degree of concordance, with a pooled Cohen’s kappa coefficient of 83% (95% CI: 79%–87%), highlighting the potential of deep learning methodologies, particularly convolutional neural networks (CNNs), to reduce interrater variability and enhance the efficiency of CAC quantification^58^.

Numerous studies further affirm the precision of AI systems in cardiovascular risk evaluation. For example, research by Lee et al. found that an AI algorithm accurately stratified cardiovascular risk in 93.9% of cases across three CT cohorts^65^. A fully automated deep learning system achieved a remarkable intraclass correlation coefficient (ICC) of 0.88 (95% CI [0.83–0.92]) when comparing ECG-gated and non-gated imaging from ^1^^8^F-fluorodeoxyglucose PET scans^66^. Additionally, Sandstedt et al. reported that AI software reached a weighted kappa of 0.919 with 89.5% accuracy in risk classification^57^. Studies such as the Framingham Heart Study further validate that deep learning systems correlate strongly with manual scoring and reliably predict cardiovascular events^56^.

The accuracy of AI-based CACS is influenced by several factors, including imaging modality, technical parameters, and the quality of the training dataset^58^. Specifically, standard CT and coronary CT angiography outperform low-dose scans, while technical specifications such as slice thickness impact efficacy. Ultimately, the diversity and quality of training data are crucial for ensuring the robustness of AI-enhanced CACS methodologies. Together, these factors underscore the valuable role of AI in modern cardiovascular risk management.

### 4.5. Influence of deep learning reconstruction and future directions for AI in CACS

Deep learning reconstruction (DLR) techniques hold significant potential for enhancing coronary artery calcium scoring (CACS) quantification by improving image quality and reducing radiation exposure, which has important implications for clinical practice. DLR algorithms effectively reduce image noise while preserving essential details, enabling lower radiation doses without compromising image quality^67^. However, studies indicate that DLR may lead to underestimation of the Agatston score, a key CACS metric, compared to traditional filtered back projection (FBP) reconstruction. This bias arises mainly from DLR’s smoothing effect, which can blur the edges of smaller calcifications, potentially resulting in failure to detect them^45,68^.

From a clinical perspective, the principal advantage of DLR is its capacity to acquire diagnostically acceptable CACS images at lower radiation doses, which is particularly valuable in minimizing radiation risks for younger patients and those requiring repeated scans. Although the potential for score underestimation remains a concern, the effect seems to be less pronounced compared to iterative reconstruction (IR) techniques^68^. It is essential for clinicians to consider these discrepancies when interpreting DLR-based CACS results.

Further research should focus on understanding the impact of different DLR strengths on CACS quantification and establishing standardized protocols for its clinical application. Clinicians should be aware of potential discrepancies between DLR-derived scores and traditional methods^45^. Looking ahead, AI technology has the potential to significantly advance CACS and cardiovascular risk assessment. Key future directions include refining AI algorithms to enhance accuracy and reliability on non-ECG-gated images, facilitating broader use of routine chest CT scans in health examinations and screening^29,61^. Furthermore, AI integration into clinical workflows can enhance reporting and cardiovascular risk profiling through the evaluation of plaque characteristics and extracoronary calcification. AI supports individualized treatment plans by enabling personalized risk prediction models incorporating genetic and imaging biomarkers. The development of explainable AI (XAI) will address the “black-box” issue, fostering clinician trust. To unlock AI’s full potential, robust validation across diverse datasets is essential, alongside clear guidelines for implementation and addressing ethical considerations such as data privacy and equitable access. With ongoing advancements, AI in CACS has the promise to transform cardiovascular risk assessment, improving efficiency and patient care^29^.

## 5. AI in CACS for risk assessment and prediction

### 5.1. Integration of coronary artery calcification scores with clinical variables

Artificial intelligence (AI) has significantly enhanced the integration of coronary artery calcification (CAC) scores with clinical variables, improving risk prediction and stratification. By combining CAC scores with demographic and clinical factors such as age, sex, lipid profiles, diabetes, and hypertension, AI models provide a more comprehensive assessment of cardiovascular risk. Emerging evidence supports the inclusion of novel biomarkers, such as epicardial adipose tissue and systemic inflammation indicators like tooth loss, in AI-driven models to refine risk prediction^69,70^. These integrations enable the identification of subtle risk patterns that traditional scoring systems may overlook.

### 5.2. Predicting cardiovascular events and outcomes

AI-based CAC scoring has demonstrated superior predictive capabilities for cardiovascular events compared to conventional risk scoring methods. A comprehensive meta-analysis revealed that deep learning algorithms could accurately predict cardiovascular outcomes, including myocardial infarction and sudden cardiac death, by analyzing imaging and clinical data^58^. Machine learning models, validated in studies published in high-impact journals, outperform traditional systems such as the Framingham Risk Score (FRS) and SCORE2 by identifying high-risk individuals with greater precision^70,71^.

Recent developments in AI-based CAC scoring have shown remarkable progress. A comprehensive meta-analysis of deep learning approaches for automated CAC scoring demonstrated that AI systems achieve high accuracy in calcium quantification. This technology is particularly valuable for ECG-gated and non-gated scans, offering flexibility in clinical applications^58^. Prior studies validated that deep learning models provide accurate and rapid coronary artery calcium scoring across different imaging protocols. These models excel in automated calcium detection, quantification of calcium burden, and risk stratification of patients^72^. AI-driven risk stratification extends to novel areas such as atrial fibrillation (AF) prediction. Studies show that integrating CAC imaging features, such as left atrial size and specific coronary calcification patterns, into machine learning algorithms improves AF risk prediction, paving the way for targeted preventive measures^70,73^.

### 5.3. Enhancing risk stratification through opportunistic screening

AI has revolutionized risk stratification by enabling opportunistic screening using non-gated and contrast-enhanced CT scans. By automating calcium scoring on non-dedicated CT protocols, AI reduces radiation exposure and costs, making cardiovascular risk assessment more accessible. These methods have demonstrated high accuracy in predicting cardiovascular events and mortality, with AI achieving an impressive accuracy of 89.4% in differentiating Agatston scores^74,75^.

### 5.4. Personalized cardiovascular risk assessments

One of AI’s most significant advantages in CAC scoring is its ability to provide personalized risk assessments. Unlike traditional models, which rely on generalized thresholds, AI dynamically adjusts predictions based on patient-specific data. AI-powered systems can recommend tailored interventions, such as lipid-lowering therapies, for patients at varying levels of risk, ensuring more effective preventive cardiology services^76^.

### 5.5. Multi-task analysis and advanced applications

AI applications in CACS extend beyond risk prediction, offering multi-task capabilities that include the simultaneous evaluation of heart failure, stroke, and non-coronary cardiac conditions^73,77^. Deep learning models have shown promising results in segment-level CAC scoring, with sensitivity and specificity rates of 73.2% and 97.8%, respectively, demonstrating their reliability in complex analyses^59^. Recent innovations have also addressed challenges such as stent filtering and the automated analysis of contrast-enhanced CT scans, broadening the clinical applicability of CAC scoring^73,74^.

### 5.6. Workflow optimization and cost-effectiveness

AI has proven to optimize clinical workflows by automating the traditionally manual and time-consuming CAC scoring process. Studies have validated the efficiency of AI algorithms, demonstrating reduced analysis time while maintaining high accuracy across diverse CT protocols^78,79^. Furthermore, AI’s ability to utilize low-dose and non-dedicated CT scans significantly reduces costs and radiation exposure, enhancing the cost-effectiveness of cardiovascular risk assessment^80^.

In conclusion, the integration of CAC scores with clinical variables through AI has transformed cardiovascular risk assessment. By incorporating demographic, clinical, and novel biomarkers, AI enhances the precision of risk prediction and stratification beyond traditional methods. Its ability to automate complex tasks, provide personalized assessments, and enable opportunistic screening underscores its potential to revolutionize preventive cardiology. While challenges such as standardization, model validation, and clinical adoption remain, the advancements in AI-driven CAC scoring hold promise for more accurate, efficient, and accessible cardiovascular care^78,79^.

## 6. AI in advancing CAC research

### 6.1. AI in analyzing large datasets for discovering new patterns and biomarkers

The application of AI in CAC research has unlocked new opportunities for analyzing large datasets, enabling the discovery of novel patterns and biomarkers. Traditional CAC scoring methods, such as the Agatston score, focus solely on calcium quantification, offering limited insights into disease mechanisms and risk prediction^81^. AI-driven approaches, however, have transformed this landscape by leveraging advanced machine learning and deep learning algorithms to extract complex features from imaging data.

Recent studies have demonstrated AI’s ability to identify previously unrecognized patterns in CAC scans. For example, machine learning systems can detect subtle calcification patterns that correlate with cardiovascular events, such as atrial fibrillation, heart failure, and stroke, expanding the scope of risk prediction^77,82^. Moreover, AI applications have facilitated the identification of novel biomarkers, including correlations between CAC patterns and systemic factors such as ACE levels, haptoglobin, and apolipoprotein AI. These insights provide a deeper understanding of coronary artery disease progression and its systemic implications^83^.

AI’s ability to integrate diverse data sources, such as imaging biomarkers and clinical variables, further enhances its role in CAC research. Advanced algorithms now combine traditional CAC scores with other factors such as epicardial adipose tissue and inflammation markers, enabling comprehensive risk stratification and personalized care^69,84^.

### 6.2. AI in accelerating clinical research and trials

AI has revolutionized the pace and efficiency of clinical research related to CAC by automating complex processes and enabling large-scale analyses. Key advancements include:

• Data Analysis Automation: AI systems can analyze vast datasets from imaging studies, significantly reducing the time required for manual interpretation. This automation allows researchers to process more data in less time, accelerating hypothesis testing and data validation^72^.
• Predictive Modeling: Machine learning models offer unparalleled capabilities in predictive analytics, facilitating the identification of high-risk populations for targeted interventions. These models enable researchers to assess the long-term impact of CAC-related variables on cardiovascular outcomes, guiding the design of clinical trials^77^.
• Improved Cohort Selection: AI-powered algorithms streamline the selection of trial participants by identifying individuals with specific CAC patterns or risk profiles. This precision reduces variability and enhances the reliability of clinical studies^85^.
• Real-time Monitoring: AI systems support real-time data analysis during trials, allowing researchers to adjust protocols dynamically based on emerging findings. This adaptability improves trial efficiency and ensures timely intervention adjustments^86^.

**Figure 2.**
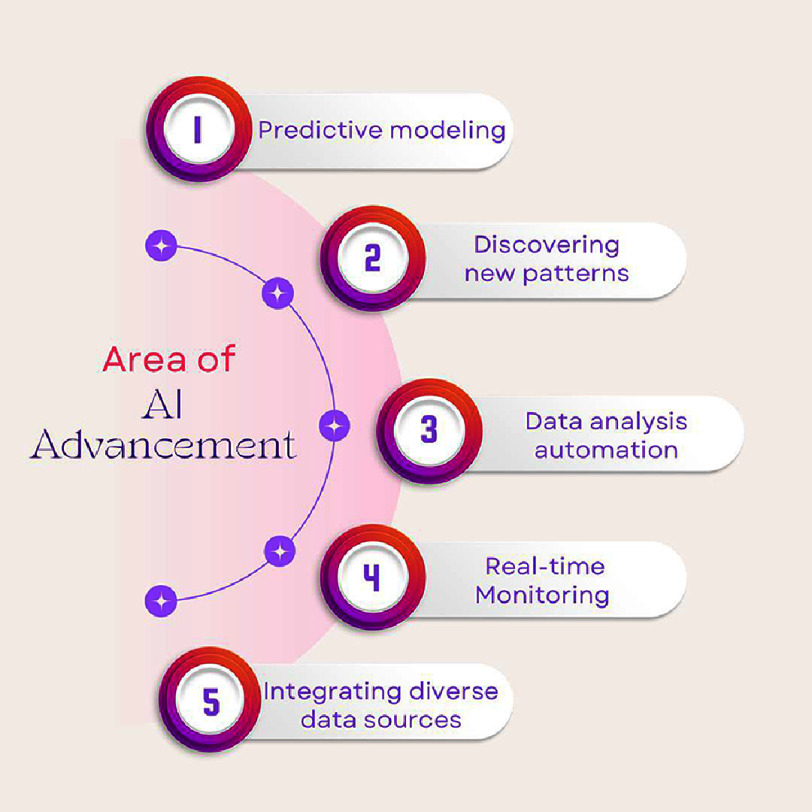
This figure illustrates the various domains of artificial intelligence advancement. 1-Identification of high-risk populations for targeted interventions. 2-Finding new patterns in CAC scans related to atrial fibrillation and novel biomarkers. 3-Analyzing large imaging datasets, speeding up manual interpretation. 4-AI enables real-time data analysis in trials. 5-AI merges CAC scores with epicardial adipose tissue and inflammation markers.

As a result, AI is revolutionizing CAC research by uncovering new patterns, biomarkers, and risk factors, thereby enhancing our understanding of coronary artery disease. Its ability to process and integrate large datasets accelerates clinical research and trials, paving the way for more targeted and efficient cardiovascular interventions. As AI continues to evolve, its role in CAC research is poised to become increasingly central to advancing cardiovascular medicine (Figure 2).

## 7. Current challenges and future perspectives

### 7.1. Challenges of AI adoption in CAC scoring

The integration of AI in healthcare, while promising, brings potential challenges related to data privacy, bias, ethics, legality, and regulations. The need to ensure patient safety, privacy, and compliance with existing healthcare standards makes it essential to address these challenges. One of the most critical issues is data privacy. AI models rely on vast amounts of medical data to function effectively, including imaging and patient records, which raises concerns about how sensitive health information is handled, particularly considering stringent regulations such as the Health Insurance Portability and Accountability Act (HIPAA) in the U.S. and the General Data Protection Regulation (GDPR) in the EU^87^. To mitigate these risks, healthcare organizations implement techniques such as encryption, access controls, and differential privacy, although anonymization remains imperfect and data sharing across institutions requires strict governance to prevent breaches or misuse^88^. Anonymizing data is a common practice to protect patient privacy, but it is not foolproof. Moreover, collaborative research often requires sharing data across institutions, which increases the risk of data breaches or misuse^87^.

Another major challenge is model bias. AI systems are only as good as the data they are trained on, and if that data is biased or unrepresentative, the AI can exhibit similar biases^89^. For instance, many medical imaging datasets used to train AI models are skewed toward specific demographics, such as white populations, while other ethnic minorities are underrepresented. Subsequently, this lack of diversity can lead to disparities in how well the model performs across different populations. Notably, the STANDING Together initiative aims to improve inclusivity and data diversity by drafting guidance for the representation and reporting of datasets, and there is a major unmet need for adoption of bias mitigation strategies in the design of AI models^90,91^. Addressing these biases requires deliberate efforts to train and validate AI models on demographically diverse datasets and incorporate algorithmic fairness techniques, alongside transparent reporting of model limitations^92^.

Finally, rigorous validation is essential before AI tools can be adopted in clinical practice. AI models must demonstrate that they are accurate, reliable, and clinically useful. However, there is no universally accepted framework for validating AI models in CAC scoring, leading to variability in model performance across different studies. Many AI models, particularly those based on deep learning, operate in a way where their decision-making processes are not easily interpretable. This lack of transparency can make clinicians hesitant to trust and adopt these tools^26^. Thus, successful clinical translation demands prospective, multicenter validation studies that assess performance across populations and imaging platforms, with thorough documentation of AI limitations and uncertainty estimates to inform clinical risk assessment. Additionally, integration challenges must be addressed, including interoperability with existing electronic health records and imaging systems, workflow adaptation, and comprehensive user training to build clinician trust and ensure safe, effective use^93^.

### 7.2. Regulatory and ethical considerations for AI in clinical practice

Despite these challenges, AI is transforming decision-support systems in healthcare, enhancing their effectiveness, efficiency, and patient-centered approach; however, it also involves navigating complex regulatory and ethical landscapes that require clear governance frameworks to ensure safety, accountability, and equitable access^94^. Within a broader framework of applying AI technologies for societal benefit, six core principles have been established in the literature to guide the ethical development of such technologies. Some of these principles are rooted in bioethics, including beneficence and non-maleficence (ensuring no harm is done and optimizing the balance between benefits and risks), autonomy (respecting individuals’ rights to make informed decisions), and justice (promoting fairness and preventing discrimination, neglect, manipulation, domination, or abuse)^95^.

Notably, the World Health Organization (WHO) in its 2021 guidance report issued a detailed framework articulating six foundational principles for AI in health, emphasizing protection of human autonomy, privacy and confidentiality, safety and efficacy, transparency, responsibility, inclusiveness, and equity to guide ethical AI development and deployment globally^94^. In a recent WHO initiative conducted in June 2021, a comprehensive set of indications, recommendations, and guidelines regarding the development, application, and utilization of AI technologies in medicine was established. The WHO work offered a detailed exploration of fundamental ethical principles designed to guide the development and implementation of AI technologies^96^. Additionally, the European Regulation enacted on April 21, 2021, categorized AI products with precision based on their potential risk to fundamental rights such as health and safety, dignity, freedom, equality, democracy, the right to be free from discrimination, and data protection^97^. The European Union’s AI Act, enacted in April 2021, introduced a risk-based regulatory framework categorizing AI systems according to their potential to affect fundamental rights and health, outlining obligations for high-risk AI systems including mandatory conformity assessments and post-market monitoring to ensure compliance with safety, transparency, and non-discrimination requirements^94^.

Accountability is one of the most important ethical challenges when developing digital technologies for healthcare. If an AI system makes an error such as misdiagnosing a patient or recommending the wrong treatment, it is not always clear who is responsible^96^. Current legal frameworks are often ill-equipped to address these issues fully, prompting calls from WHO and regulatory bodies for new guidelines and governance mechanisms that clearly delineate accountability across developers, deployers, healthcare providers, and regulators to manage AI-related risks effectively^94^.

Finally, over-reliance on technology represents another potential threat. While AI can enhance clinical decision-making, there is a risk that clinicians may become too dependent on these tools, potentially overlooking their own expertise or failing to recognize when the AI’s output is flawed. WHO highlights this “automation bias” and underscores the critical need for maintaining clinician autonomy and accountability as AI tools are integrated into healthcare, recommending appropriate training and transparent communication of AI system limitations to ensure balanced human-machine decision-making^94^. Achieving the correct balance between human clinical judgment and machine assistance is a key success factor to ensure that AI is used as a tool to support, rather than replace, clinicians^98^.

### 7.3. Future advancements in AI for CAC scoring

The future of AI in coronary artery calcium (CAC) scoring is incredibly promising, with several advancements in how clinicians could approach cardiovascular risk assessment. One of the most immediate benefits of AI is speed. Traditional CAC scoring requires radiologists to manually identify and quantify calcified plaques in coronary arteries, which can be time-consuming. AI-powered deep learning algorithms are already showing the ability to analyze CT scans in seconds. This speed could significantly improve workflow efficiency, especially in high-volume clinical care settings^45^.

AI-powered deep learning algorithms, such as convolutional neural networks (CNNs), have revolutionized medical imaging. A meta-analysis conducted by Wang et al. in 2024 demonstrated the effectiveness of deep learning models in automating CAC scoring and quantification of coronary calcification, achieving a high level of accuracy and consistency. The findings underscore the potential of these models to streamline the CAC scoring process, reduce interrater variability, and enhance cardiovascular risk prediction. The results show strong agreement between deep learning models and manual scoring methods, but further refinement is needed to improve model generalizability across diverse patient populations and imaging protocols^58^.

Beyond speed and accuracy, AI enables enhanced harmonization of results across different institutions. The lack of reproducibility due to variability between readers and institutions has long been a challenge in CAC scoring. AI can standardize the process, ensuring that CAC scores are consistent. AI models trained on multi-institutional datasets have achieved remarkable reproducibility, reducing inter-observer variability^58^.

To fully realize these benefits, future research priorities should focus on:

• Developing large-scale, diverse, multicenter datasets that include representative samples from varied demographics, scanner types, and clinical settings to enhance AI model robustness and generalizability^58^.
• Establishing internationally recognized standards and protocols for CAC scoring with AI, including data acquisition, annotation, and reporting formats, to reduce heterogeneity and enable consistent comparisons across studies, clinical trials, and routine practice.
• Designing prospective clinical trials to assess the real-world impact of AI-assisted CAC scoring on clinical decision-making, patient outcomes, and cost-effectiveness.
• Exploring integration strategies to seamlessly embed AI CAC scoring tools into clinical workflows and electronic health record systems, with attention to user interface design and clinician training^99^.

Standardization remains critical to minimize variability across imaging centers and to enable use of AI-powered CAC scores for longitudinal risk surveillance and therapeutic guidance. Collaborative efforts by professional societies, regulatory agencies, and industry stakeholders are required to develop consensus guidelines and validation benchmarks^45,58^.

Regarding timeline expectations, given the current pace of development and initial FDA clearances of AI tools in cardiovascular imaging, broader clinical implementation of reliable AI-based CAC scoring tools may be expected within the coming years. However, full integration with clinical practice will depend on demonstrating sustained accuracy in diverse populations, regulatory approvals, reimbursement policies, and clinician acceptance^58^.

### 7.4. AI integration with emerging health technologies

Wearable devices such as smartwatches and fitness trackers have become incredibly advanced, capable of tracking heart rate, rhythm, blood pressure, sleep patterns, and even oxygen saturation. The integration of AI into these devices is transformative, revolutionizing cardiovascular care. Beyond enabling early detection of conditions that might otherwise go unnoticed, AI-powered wearables facilitate real-time monitoring and the creation of personalized risk profiles. By combining data from wearables with electronic health records (EHRs), genetic information, and environmental factors, these tools empower clinicians to shift from a reactive to a proactive approach in patient care^100^.

Notably, AI shows great promise in remote patient monitoring and telemedicine. It can even serve as a virtual health coach, encouraging patients to take their medications, stay active, or adopt healthier eating habits, all based on data collected from their wearables. This capability is especially valuable for fostering long-term behavior change, which plays a crucial role in maintaining and improving cardiovascular health^101^. However, successful integration of these wearable AI tools into clinical workflows requires addressing challenges related to data interoperability, patient privacy, and the need for clinicians to interpret AI-derived insights alongside clinical judgment to avoid over-reliance on automated outputs^102^.

### 7.5. The role of CT-FFR in combination with AI and CACS

The combination of CT-derived fractional flow reserve (CT-FFR), AI, and coronary artery calcium (CAC) scoring represents a powerful toolkit for diagnosing and managing coronary artery disease (CAD). When combined, these technologies provide a more comprehensive and personalized evaluation of cardiovascular risk, integrating anatomical and functional information^103^. CT-FFR is a non-invasive technique that uses coronary computed tomography angiography (CCTA) images to estimate the functional significance of coronary artery lesions. Traditionally, the relevance of coronary stenoses was assessed using fractional flow reserve (FFR) measured during a catheterization procedure^104^.

AI enhances CT-FFR by automating the analysis of CCTA images, creating 3D models of the coronary arteries, and simulating blood flow. This reduces the time needed for analysis from hours to minutes and improves accuracy by learning from vast datasets of CCTA images and invasive FFR measurements^105^. Despite these advances, clinical adoption of AI-enhanced CT-FFR tools requires robust prospective validation, integration with electronic health records, and user training to ensure safe and effective interpretation of results by multidisciplinary teams^103^.

AI further enhances this synergy by integrating data from CAC scoring and CT-FFR to provide a more personalized risk assessment^106^.

These integrated AI-empowered tools, while promising, must still undergo rigorous clinical validation and adoption pathways to ensure accuracy, reproducibility, and safety. Their effective clinical implementation also depends on seamless integration with patient health records and clinician workflows, along with clear guidelines on interpretation and use within multidisciplinary care teams. Ongoing research is needed to standardize these combined AI approaches and to define clear clinical guidelines to support clinician decision-making.

## 8. Limitations

This narrative review has several limitations that should be acknowledged. First, although we aimed to include the most relevant and up-to-date literature on artificial intelligence in coronary artery calcium (CAC) scoring, the selection of studies was limited to publications in English and those indexed in PubMed, ScienceDirect, and Semantic Scholar. This language and database restriction may introduce selection bias, potentially excluding valuable research published in other languages or in non-indexed sources.

Second, as this is not a systematic review, we did not perform a formal quality assessment of included studies using standardized tools. Instead, we relied on the peer-reviewed status and perceived relevance of each study. This approach may limit the reproducibility and comprehensiveness of the findings.

Third, the heterogeneity of study designs, AI models, imaging modalities, and outcome measures across the included literature made direct comparison difficult and precluded quantitative synthesis. Therefore, our findings are meant to provide a broad overview and qualitative insight rather than definitive evidence-based conclusions.

## 9. Conclusion

The use of artificial intelligence (AI) in coronary artery calcification (CAC) scoring signifies a significant advance in cardiovascular risk evaluation. Our study indicates that AI techniques, especially deep learning and machine learning, have proven highly effective in enhancing the accuracy and consistency of CAC assessment. AI can play a critical role in facilitating prompt interventions and enhancing patient outcomes through precise assessment. However, despite these encouraging advancements, numerous challenges persist. The requirement for validating AI algorithms across diverse populations is crucial to ensure generalizability and equity in healthcare delivery. Furthermore, properly integrating AI tools into routine clinical practice requires overcoming constraints such as physician training, workflow compliance, and regulatory considerations.

Looking ahead, further research should focus on developing user-friendly AI applications that are easily accessible to healthcare professionals and expand their applicability in everyday practice. Furthermore, exploring the use of AI in longitudinal studies of cardiovascular health may provide deeper insights into disease progression and patient management. By overcoming existing challenges and harnessing the full potential of AI, we can improve CAC scoring, ultimately leading to more personalized and effective cardiovascular care.

## Author contributions

**Conceptualization:** Mobin Ghaderi, Reza Golchin Vafa. **Methodology:** Mobin Ghaderi, Reza Golchin Vafa. **Writing – Original Draft Preparation:** Mobin Ghaderi, Reza Golchin Vafa, Najmeh Vosoughiyan, Sultan Mujib Dabiry, Omneya Abdelkarem. **Writing – Review & Editing:** Mobin Ghaderi, Najmeh Vosoughiyan, Sultan Mujib Dabiry, Omneya Abdelkarem. **Investigation:** Mobin Ghaderi, Reza Golchin Vafa, Sultan Mujib Dabiry, Omneya Abdelkarem. **Visualization:** Najmeh Vosoughiyan, Sultan Mujib Dabiry. **Supervision:** Mobin Ghaderi.

## Acknowledgments

Artificial intelligence in the form of ‘QuillBot’ has been used to improve the language.

## Conflicts of interest

The author has no potential conflicts of interest to disclose.
